# Cartilage Derived from Bone Marrow Mesenchymal Stem Cells Expresses Lubricin *In Vitro* and *In Vivo*

**DOI:** 10.1371/journal.pone.0148777

**Published:** 2016-02-11

**Authors:** Yusuke Nakagawa, Takeshi Muneta, Koji Otabe, Nobutake Ozeki, Mitsuru Mizuno, Mio Udo, Ryusuke Saito, Katsuaki Yanagisawa, Shizuko Ichinose, Hideyuki Koga, Kunikazu Tsuji, Ichiro Sekiya

**Affiliations:** 1 Department of Joint Surgery and Sports Medicine, Graduate School, Tokyo Medical and Dental University, Tokyo, Japan; 2 Center for Stem Cell and Regenerative Medicine, Tokyo Medical and Dental University, Tokyo, Japan; 3 Research Center for Medical and Dental Sciences, Tokyo Medical and Dental University, Tokyo, Japan; 4 Department of Cartilage Regeneration, Tokyo Medical and Dental University, Tokyo, Japan; University of Alabama at Birmingham, UNITED STATES

## Abstract

**Objective:**

Lubricin expression in the superficial cartilage will be a crucial factor in the success of cartilage regeneration. Mesenchymal stem cells (MSCs) are an attractive cell source and the use of aggregates of MSCs has some advantages in terms of chondrogenic potential and efficiency of cell adhesion. Lubricin expression in transplanted MSCs has not been fully elucidated so far. Our goals were to determine (1) whether cartilage pellets of human MSCs expressed lubricin *in vitro* chondrogenesis, (2) whether aggregates of human MSCs promoted lubricin expression, and (3) whether aggregates of MSCs expressed lubricin in the superficial cartilage after transplantation into osteochondral defects in rats.

**Methods:**

For *in vitro* analysis, human bone marrow (BM) MSCs were differentiated into cartilage by pellet culture, and also aggregated using the hanging drop technique. For an animal study, aggregates of BM MSCs derived from GFP transgenic rats were transplanted to the osteochondral defect in the trochlear groove of wild type rat knee joints. Lubricin expression was mainly evaluated in differentiated and regenerated cartilages.

**Results:**

In *in vitro* analysis, lubricin was detected in the superficial zone of the pellets and conditioned medium. mRNA expression of Proteoglycan4 (Prg4), which encodes lubricin, in pellets was significantly higher than that of undifferentiated MSCs. Aggregates showed different morphological features between the superficial and deep zone, and the Prg4 mRNA expression increased after aggregate formation. Lubricin was also found in the aggregate. In a rat study, articular cartilage regeneration was significantly better in the MSC group than in the control group as shown by macroscopical and histological analysis. The transmission electron microscope showed that morphology of the superficial cartilage in the MSC group was closer to that of the intact cartilage than in the control group. GFP positive cells remained in the repaired tissue and expressed lubricin in the superficial cartilage.

**Conclusion:**

Cartilage derived from MSCs expressed lubricin protein both *in vitro* and *in vivo*. Aggregation promoted lubricin expression of MSCs *in vitro* and transplantation of aggregates of MSCs regenerated cartilage including the superficial zone in a rat osteochondral defect model. Our results indicate that aggregated MSCs could be clinically relevant for therapeutic approaches to articular cartilage regeneration with an appropriate superficial zone in the future.

## Introduction

Articular cartilage has a highly organized structure composed of four zones: superficial zone, middle zone, deep zone and calcified zone [1, 2]. The chondrocyte phenotype, cell shape, and the extracellular matrix structure vary among the different zones. Osteoarthritis (OA) is a joint disease that mostly affects cartilage. OA is a multifactorial disease with various etiologies involving hereditary, age-related, post-traumatic, mechanical, and other factors [3, 4]. The disease progression caused loss of proteoglycan, disruption of the collagen network in the articular cartilage, and alternations in other joint tissues such as the meniscus, synovium, and subchondral bone [5, 6]. OA is the most common form of arthritis, and extracellular matrix degeneration generally begins in the superficial zone of the articular cartilage, and progresses to deeper zones [7].

The superficial cartilage contains a mucinous glycoprotein, lubricin, encoded by the proteoglycan 4 (Prg4) gene [8]. Lubricin functions as a boundary lubricating molecule on the surfaces of articular cartilage to reduce friction and wear [9–11]. An increasing number of papers demonstrated that the lubricin expression level decreased in various OA animal models [12, 13] and in human patients following anterior cruciate ligament injury, a significant risk factor for OA [14]. Furthermore, Prg4 gene mutation causes camptodactyly-arthropathy-coxa vara-pericarditis syndrome in humans. Patients with this disorder develop a non-inflammatory arthritis, which leads to joint failure at an early age [15], and Prg4 knockout mice develop premature cartilage degeneration [16]. Based on these findings, lubricin has chondroprotective effects and plays a pivotal role in maintaining articular cartilage homeostasis. Thus, clinicians and researchers have begun to pay more attention to lubricin because a better understanding of the role of lubricin would lead to improved treatment of OA [17, 18]. Recently, various approaches to augmenting lubricin expression in the joint cavity were applied to ameliorating OA, such as supplementation of recombinant lubricin [19–22], Prg4 overexpression [23], mechanical stimulation (exercise) [24–26], and diet such as extra-virgin olive oil [27] in the animal model of OA. Regarding articular cartilage regeneration, increasing lubricin expression in the superficial cartilage will be an important factor as well [28]. Thus, cell sources require both a chondrocytic and lubricin-producing phenotype. Clinically, various methods have been applied to regenerating articular cartilage, such as autologous chondrocyte implantation [29, 30]; however, it is still difficult to regenerate superficial cartilage so far.

Mesenchymal stem cells (MSCs) are an attractive cell source and the use of aggregates of MSCs has some advantages for cartilage regeneration [31–34]. Aggregation of MSCs enhanced chondrogenic potential, and increased the number of MSCs attached to the defect, and MSCs survived longer after transplantation of aggregates of MSCs in comparison to suspension transplantation of MSCs [34, 35]. The use of aggregates is also practically convenient for cartilage regeneration with MSCs in that aggregates of MSCs are easily formed, visible, and solid enough to aspirate with a syringe. There are some reports describing that MSCs derived from bone marrow (BM) [28], synovium [36], adipose tissue [37], and muscle [38] expressed lubricin in specific conditions *in vitro*. However, lubricin expression in transplanted MSCs has not been fully elucidated so far. In this study, we examined (1) whether cartilage pellets of human MSCs expressed lubricin after *in vitro* chondrogenesis, (2) whether aggregates of human MSCs promoted lubricin expression after hanging drop culture, and (3) whether aggregates of MSCs expressed lubricin in the superficial cartilage after transplantation into osteochondral defects in rats. We hypothesized that MSCs were able to differentiate into the phenotype of the superficial zone chondrocyte that expressed lubricin in both *in vitro* and *in vivo* chondrogenesis, and that transplantation of aggregates of MSCs enhanced cartilage regeneration with an appropriate superficial zone.

## Materials and Methods

Human bone marrow (BM) MSCs were differentiated into cartilage by pellet culture and hanging drop techniques. Additionally, aggregates of GFP transgenic rat BM MSCs were transplanted to the osteochondral defect in wild type rats. Lubricin expression was mainly evaluated in differentiated and regenerated cartilages.

### Isolation and Culture of Human BM MSCs

The study was approved by the local Institutional Review Board of Tokyo Medical and Dental University (No.1030), and full written informed consent was obtained from all donors included in this study prior to the operative procedure. Bone marrow from the tibia was obtained with an 18-gauge needle during knee operations. Nucleated cells from the bone marrow were isolated with a density gradient (Ficoll-Paque; Amersham Biosciences, Uppsala, Sweden). Nucleated cells were plated at a clonal density in complete culture medium (CCM: αMEM) (Invitrogen, Carlsbad, CA) containing 10% fetal bovine serum, 100 units/ml penicillin, 100 mg/ml streptomycin, and 250 ng/ml amphotericin B (Invitrogen) for 14 days. Passage 2 MSCs were used for the following analyses.

### *In Vitro* Chondrogenesis with Pellet Culture

2.5 × 10^5^ human BM-MSCs were pelleted, centrifuged, and cultured in 400 μl chondrogenic medium (high-glucose Dulbecco's modified Eagle's medium [Invitrogen], 10^−7^ M dexamethasone, 50 μg/ml ascorbate-2-phosphate, 40 μg/ml proline, 100 μg/ml pyruvate, and 50 mg/ml ITS^™^ Premix [BD Falcon, Franklin Lakes, NJ]) supplemented with 10 ng/ml TGF-β3 (R&D Systems, Minneapolis, MN) for 21 days.

### Histological Analysis for Cartilage Pellets

For making a paraffin section, the cartilage pellet was fixed in 4% paraformaldehyde for 1 day, then embedded in paraffin wax. The specimens were cut into 5 μm thick sections. Histological pictures were taken by an Olympus IX71 light microscope (Olympus, Tokyo, Japan). The cartilage pellet was divided into two zones, superficial and deep zone, by the difference of cell morphology and extra cellular matrix stained with Safranin-O/Fast Green [39]. The percentage of lubricin positive cells was measured for extent score [37] as a percentage in five points as follows: 0 = 0–5%; 1 = 5–30%; 2 = 31–50%; 3 = 51–75%; 4 = >75%. These scores were evaluated by two independent observers in a blinded manner.

### Immunostaining of Lubricin for Cartilage Pellets Derived from Human MSCs

Paraffin-embedded sections were deparaffinized and heated by a microwave oven in citrate buffer (pH6) (Dako, Glostrup, Denmark), and endogenous peroxidases were quenched using 0.3% hydrogen peroxidase in methanol for 15 minutes. A rabbit polyclonal human anti-lubricin antibody (1:200 dilution; ab28484 Abcam, Cambridge, England) was applied to sections and incubated at room temperature for 1 hour. The sections were incubated in the biotinylated goat anti-rabbit IgG (1:200 dilution; Vector, Burlingame, CA) for 30 minutes at room temperature. Immunostaining was detected with Vectastain ABC regent (Vector) followed by diaminobenzidine staining. The sections were counterstained with Hematoxylin. Negative controls were incubated without the primary antibody.

### Transmission Electron Microscopy

The specimens of the aggregate cultured for 3 days, the cartilage pellet cultured for 21 days, and the dissected femoral condyles were fixed in 2.5% glutaraldehyde in 0.1M phosphate buffer for 2 hours. The samples were washed with 0.1M phosphate buffer, post-fixed in 1% OsO4 buffered with 0.1M phosphate buffer for 2 hours, dehydrated in a graded series of ethanol and embedded in Epon 812. Semi-thin sections at 1 μm were collected on glass slides and stained for 30 seconds with Toluidine Blue and then examined by a light microscope. Ultrathin sections at 90 nm were collected on copper grids, double-stained with uranyl acetate and lead citrate, and then examined by transmission electron microscopy (H-7100, Hitachi, Tokyo, Japan) [40].

### RT-PCR

Total RNA was extracted from human bone marrow MSCs in a monolayer culture, the aggregates were cultured for 3 days, and the pellets cultured for 7 days using a High Pure RNA Isolation kit (Roche Applied Sciences, Mannheim, Germany) after aggregates and pellets were digested with 3 mg/ml collagenase. First-strand complementary DNAs (cDNAs) were synthesized with oligo-dT primers from total RNA using the Transcriptor High Fidelity cDNA Synthesis Kit (Roche Diagnostics, Mannheim, Germany). RT-PCR was performed in a LightCycler 480 instrument (Roche Diagnostics) with up to 45 cycles using FastStart TaqMan Probe Master and TaqMan probes (Roche Diagnostics). The primers and TaqMan probes were as shown in S1 Table. The amounts of mRNA were calculated as relative quantities in comparison to β-actin mRNA. Ct value of 35 was considered as minimum gene expression [41].

### Western Blot

Conditioned medium for pellet culture between 17 and 21 days and conditioned medium for monolayer culture were collected. 10 μl medium was loaded on each lane separated by electrophoresis with 4–20% gel (Invitrogen). Proteins were transferred onto nitrocellulose membranes by semi wet blotting. Membranes were blocked in 5% skim milk at 4°C overnight and incubated with a rabbit polyclonal anti-lubricin antibody (1:100 dilution; H-140, Santa Cruz Biotechnology, Santa Cruz, CA) at 4°C overnight. Antibodies were detected with horseradish peroxidase-conjugated secondary antibody (Millipore, Billerica, MA) using the enhanced chemiluminescent detection ECL-Prime (GE Healthcare, Little Chalfont, UK).

### Preparation of Aggregate of BM MSCs

2.5 × 10^5^ human MSCs were prepared, resuspended in 35 μl of CCM, and plated on an inverted culture dish lid. Then, the lid was inverted and placed on a culture dish. The cells were cultured for 3 days in hanging drops [34, 35].

### Animals

This animal study was approved by the Animal Experimentation Committee of Tokyo Medical and Dental University (No.0140222, No.0150308), and animal care and all experiments were conducted in accordance with guidelines. Thirty-four wild type female Lewis rats at ten to fourteen weeks were purchased from Charles River Laboratories Japan (Kanagawa, Japan). Two green fluorescence protein expressing transgenic Lewis rats were provided by Jichi Medical University (Tochigi, Japan) [42]. Rats were housed in a standardized cage under a 12 hour light–dark cycle and allowed food and water ad libitum.

### Isolation and Culture of Rat BM MSCs

Bone marrow was extruded by inserting a 22-gauge needle into the shaft of the femur and tibia bone of 2 GFP transgenic Lewis rats and was then flushed out. The following procedures were as mentioned above. Passage 2 MSCs were used for the following analyses.

### Transplantation of Aggregates to Osteochondral Defect in Rats

Wild type female Lewis rats were used as recipients, and GFP transgenic Lewis rats were used as donors in this cell transplantation experiment. After intraperitoneal injection of 500 mg/kg tribromoethanol and under 2.5% isoflurane inhalation, the knee joint was approached through a medial parapatellar incision, and the patella was dislocated laterally. Full-thickness osteochondral defects (diameter 1.5 mm, 1.0 mm deep) were created in the trochlear groove of the femur. For the MSC group, 3 days before surgery, passage 2 GFP positive MSCs were aggregated by hanging drop culture. Four aggregates of GFP rat BM MSCs were transplanted to the defect. For the control group, the defect was left empty. After surgery, rats were allowed to be active without any fixation device or immobilization. Animals were harvested with CO_2_ inhalation at 1 hour (n = 1 in each group), 4 weeks (n = 8 in each group) and at 12 weeks (n = 8 in each group) after the operation.

### Macroscopic Examination

The dissected femoral condyle was observed macroscopically under a bright field and fluorescence for GFP using an Olympus MVX10 stereoscopic microscope (Olympus) to examine repaired tissue. Repaired tissues were evaluated using a macroscopic score based on the following tissue color grading: 1-depression (tissue void), 2-red/grey, 3-beige, 4-red-white, 5-white homogeneous tissue [43].

### Histological Examination *In Vivo*

The dissected femoral condyle was fixed in 4% paraformaldehyde for 7 days, decalcified in 20% EDTA solution for 21 days, then embedded in paraffin wax. The specimens were sectioned in the sagittal plane at 5 μm. Histological sections of the repaired tissue stained with Safranin-O/Fast Green were analyzed using a grading system that was modified from the repaired cartilage score described by Wakitani (S2 Table) [44]. These scores were evaluated by two independent observers in a blinded manner. Unstained slides were observed with an Olympus BX51 polarizing microscope (Olympus) in order to evaluate regularity of collagen fiber orientation [45, 46]. The intact articular cartilage was not operated.

### Immunostaining of Lubricin for Regenerated Cartilage in Rats

The dissected femoral condyle was immediately embedded in Super Cryo Embedding Medium (Section-lab, Co., Ltd., Hiroshima, Japan) and frozen with dry ice and hexane. Cryosections at 12 μm were prepared with a CM 3050S cryostat (LEICA, Nussloch, Germany) and an ultracut S microtome (Reichert, Vienna, Austria). Rabbit polyclonal anti-lubricin antibody H-140 (1:100 dilution; H-140, Santa Cruz Biotechnology) was applied to sections and incubated at 4°C overnight. Goat anti-mouse IgG labeled with Alexa fluor 555 (1:500 dilution, Invitrogen) was applied and incubated for 1 hour at room temperature. Background nuclei were counterstained with Hoechst 33342 (Invitrogen). Negative controls were incubated without the primary antibody. The specimens were photographed under the condition of fluorescence using Olympus MVX10.

### Immunostaining for GFP

Paraffin-embedded sections were deparaffinized and pretreated with peroxidase K (Dako, Glostrup, Denmark) in Tris HCL buffer at room temperature, and endogenous peroxidases were quenched using 0.3% hydrogen peroxidase in methanol for 15 minutes. Rabbit polyclonal anti-GFP antibody (1:100 dilution; A01704-100, GenScript, Piscataway, NJ) was applied to sections and incubated at room temperature for 1 hour. The sections were incubated in the biotinylated goat anti-rabbit IgG (1:200 dilution; Vector, Burlingame, CA) for 30 minutes at room temperature. Immunostaining was detected with Vectastain ABC regent (Vector) followed by diaminobenzidine staining. The sections were counterstained with Hematoxylin.

### Micro Computed Tomography Scanning

The dissected femoral condyles were subjected to analysis using a high-resolution micro computed tomography scanner (ScanXmate-E090; Comscantecno, Kanagawa, Japan). The images were reconstituted using TRI/3D-BON software (RATOC, Tokyo, Japan).

### Statistical Analyses

Comparisons between two groups were analyzed using the Wilcoxon’s signed rank test in *in vitro* study and the Mann-Whitney U test in *in vivo* study. P-values less than 0.05 were considered to be statistically significant.

## Results

### Lubricin Expression in Human BM MSCs after Pellet Culture

**Fig 1:**
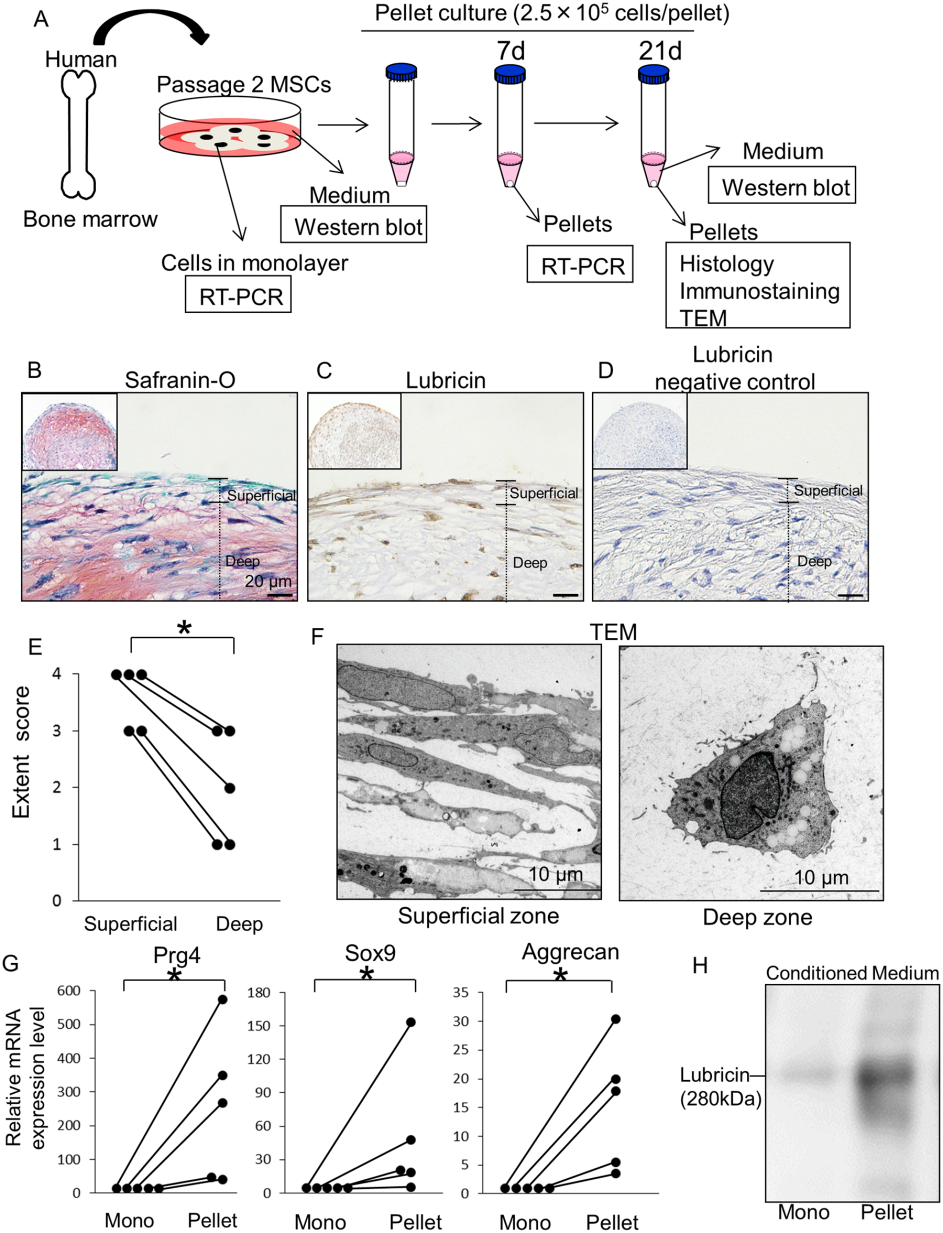
Lubricin expression in human BM MSCs after pellet culture. (A) Schema for the study of *in vitro* chondrogenesis. BM MSCs were prepared from 5 donors. 2.5 × 10^5^ human BM MSCs at passage 2 were pelleted and cultured in chondrogenic medium for 7 or 21 days. (B) Histological sections of cartilage pellets at day 21 stained with Safranin-O/Fast Green. (C) Cartilage pellets immunostained with lubricin. (D) Cartilage pellets immunostained without primary lubricin antibody as negative control. (E) Extent score for lubricin positive cell rate [37] (n = 5, *p<0.05 by the Wilcoxon’s signed rank test). (F) Transmission electron microscopy (TEM) images for pellets at 21 days. (G) Real time PCR analysis for MSCs 7 days after monolayer (Mono) and pellet culture (Pellet). RNA was prepared from 500,000 MSCs or 2 pellets from 5 donors respectively. Gene expression levels in MSCs after monolayer culture were normalized as 1. (H) Western blotting image for lubricin protein of medium obtained 21 days after monolayer and pellet culture.

Cells derived from human BM had characteristics of MSCs indicated by colony formation, multipotentiality, and surface epitopes (S1 Fig). For *in vitro* chondrogenesis analysis, pelleted MSCs were cultured in chondrogenic medium supplemented with 10 ng/ml TGF-β3 up to 21 days (Fig 1A). Pellets showed chondrogenic phenotype indicated by Safranin-O/Fast Green staining (Fig 1B). Interestingly, the superficial zone of pellets consisting of the flattened cells was not stained with Safranin-O. Lubricin protein was also observed in pellets (Fig 1C and 1D). To analyze lubricin distribution in the pellet, lubricin positive cell rate was measured in the superficial and deep zone. The extent score in the superficial zone was significantly higher than that in the deep zone (Fig 1E). Transmission electron microscopic images showed spindle-shape cells with an ovoid nucleus in the superficial zone and chondrocyte-like cells producing a large amount of matrix vesicles in the deep zone (Fig 1F). A recognizable lamina splendens was not seen in the surface of pellets. RT-PCR analysis showed that the Prg4 mRNA expression in pellets increased at 7 days, along with chondrogenesis related genes, Sox9 and Aggrecan (Fig 1G). Western blot analysis showed higher lubricin protein expression in the conditioned medium after pellet culture than after monolayer culture (Fig 1H).

### Morphology and Gene Expressions of Aggregate of Human BM MSCs

**Fig 2:**
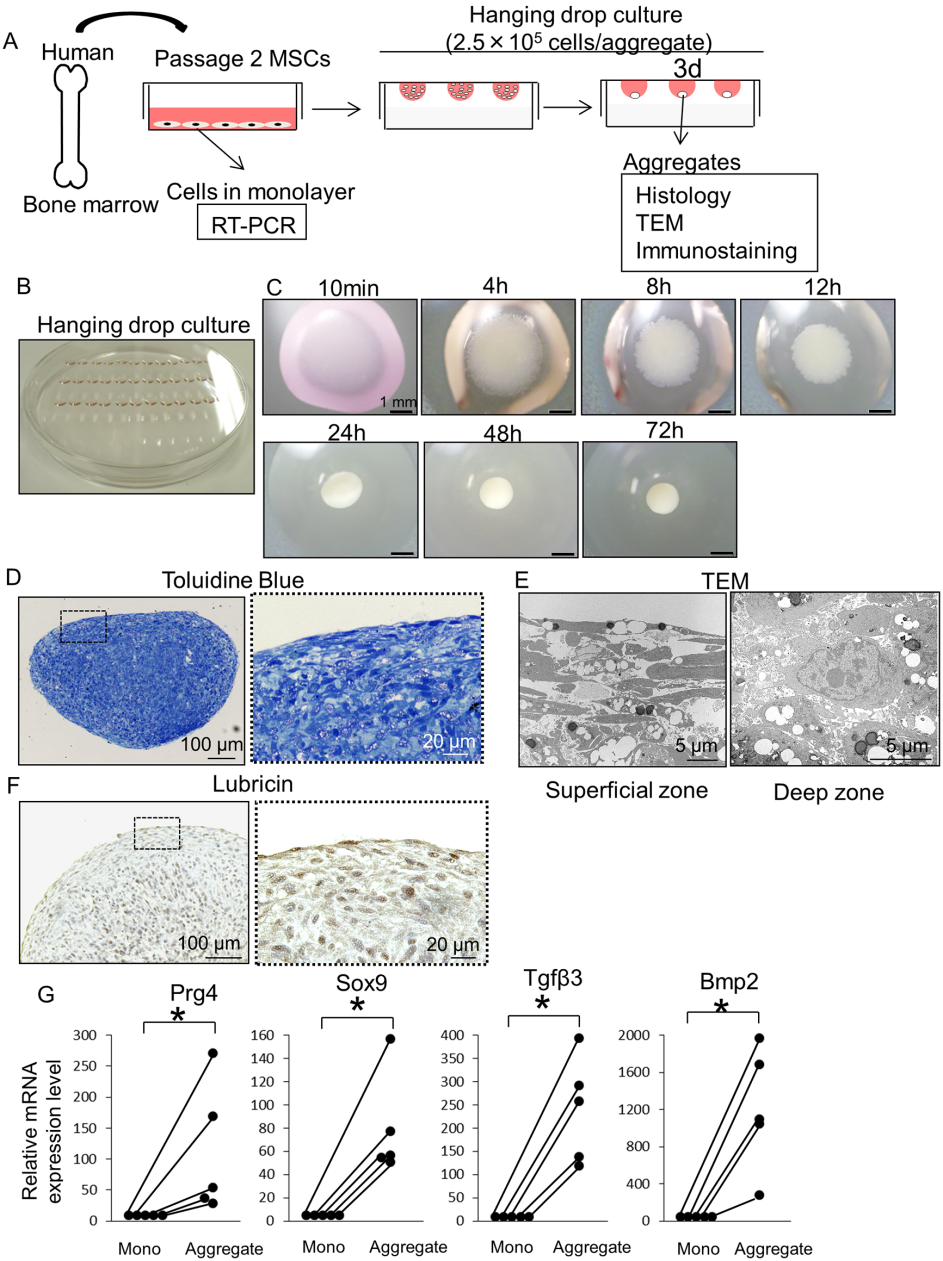
Preparation, appearance and gene expressions of aggregates of human BM MSCs after hanging drop culture. (A) Schema for the study. 2.5 × 10^5^ human BM MSCs were cultured for 3 days in hanging drops. (B) Drops hanging on the cover of a 15 cm dish. (C) Macroscopic images of aggregates consisting of 2.5×10^5^ MSCs at 10 minutes-72 hours. (D) Histological sections stained with Toluidine Blue at 72 hours. (E) TEM images of aggregates at 72 hours. (F) Histological sections immunostained with lubricin at 72 hours. (G) Real time PCR analysis for MSCs 72 hours after monolayer and pellet culture. RNA was prepared from 500,000 MSCs or 2 aggregates from 5 donors respectively. Gene expression levels in MSCs after monolayer culture were normalized as 1 (n = 5, *p<0.05 by the Wilcoxon’s signed rank test).

Human BM MSCs were aggregated using the hanging drop technique (Fig 2A and 2B). MSCs cultured in the drop gradually condensed, and the aggregate consisting of 2.5 × 10^5^ MSCs became approximately 1 mm in diameter 72 hours after hanging drop culture (Fig 2C). Sagittal sections of the aggregate showed an oval round-shape as a whole (Fig 2D). The superficial zone was composed of spindle cells parallel to the surface, whereas the deep zone was composed of round cells (Fig 2D and 2E). Lubricin protein was found in the aggregates (Fig 2F). mRNA expressions for Prg4, Sox9, Tgf-β3, and Bmp2 in MSCs increased 72 hours after hanging drop culture (Fig 2G).

### Cartilage Regeneration by Aggregates of BM MSCs in Rats

**Fig 3:**
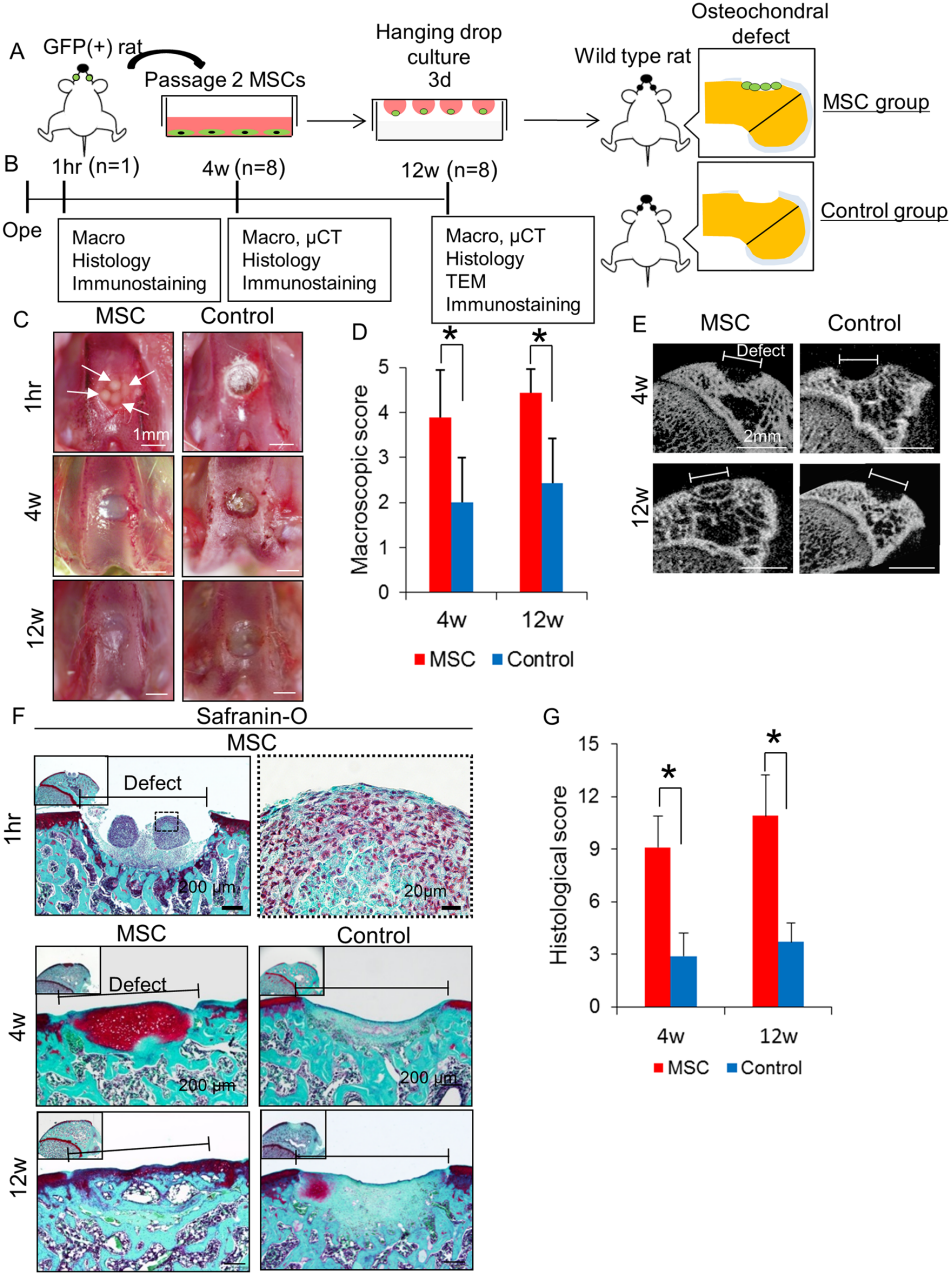
Cartilage regeneration by transplantation of aggregates of BM MSCs in rats. (A) Schema for the study. BM MSCs were obtained from GFP transgenic rats. Three days before the surgery, passage 2 GFP positive MSCs were aggregated by hanging drop culture. Osteochondral defect was created in the knee joint. For the MSC group, 4 aggregates were transplanted to the defect. For the control group, the defect was left empty. (B) Protocol for evaluation. (C) Macroscopic observation of osteochondral defects after aggregate transplantation. For the MSC group, 4 aggregates were transplanted to the defect. For the control group, the cartilage defect was left empty. White arrows indicate aggregates of BM MSCs. (D) Macroscopic score for cartilage [43]. Averaged score with standard deviation is shown (n = 7, *p<0.05 by the Mann–Whitney U test). (E) CT images. (F) Sagittal sections stained with Safranin-O/Fast Green. (G) Histological score by Wakitani et al. (S2 Table) [44]. Averaged score with standard deviation is shown (n = 7, *p<0.05 by the Mann–Whitney U test).

For *in vivo* analysis, aggregates consisting of MSCs derived from BM of a GFP transgenic rat were transplanted onto osteochondral defects in rats (Fig 3A). Animals were analyzed 1 hour, 4 weeks and 12 weeks after surgery (Fig 3B). The GFP cells had characteristics of MSCs (S2 Fig). Macroscopically, at 4 weeks, the defect became whitish and glossy in the entire area in the MSC group, while the peripheral area of the defect was still reddish in the control group (Fig 3C). At 12 weeks, the border between repaired tissue and adjacent cartilage appeared less distinct in the MSC group, whereas the defect was occupied with beige tissue in the control group. The macroscopic score in the MSC group was significantly superior to that in the control group both at 4 and 12 weeks (Fig 3D). μCT images revealed that subchondral bone was regenerated in the MSC group but not in the control group at 12 weeks (Fig 3E). Histologically at 1 hour, aggregates were confirmed on the defect (Fig 3F). At 4 weeks in the MSC group, the osteochondral defect was filled with abundant cartilage matrix stained with Safranin-O/Fast Green, whereas in the control group, cartilage matrix formation appeared poor. At 12 weeks, in the MSC group, the border between cartilage and subchondral bone moved upward and the thickness of the regenerated cartilage became similar to the adjacent one. In the control group, the defect had still healed poorly. The histological score in the MSC group was significantly higher than that in the control group at both 4 and 12 weeks (Fig 3G).

### Histological Analysis of the Superficial Zone in Regenerated Cartilage

**Fig 4:**
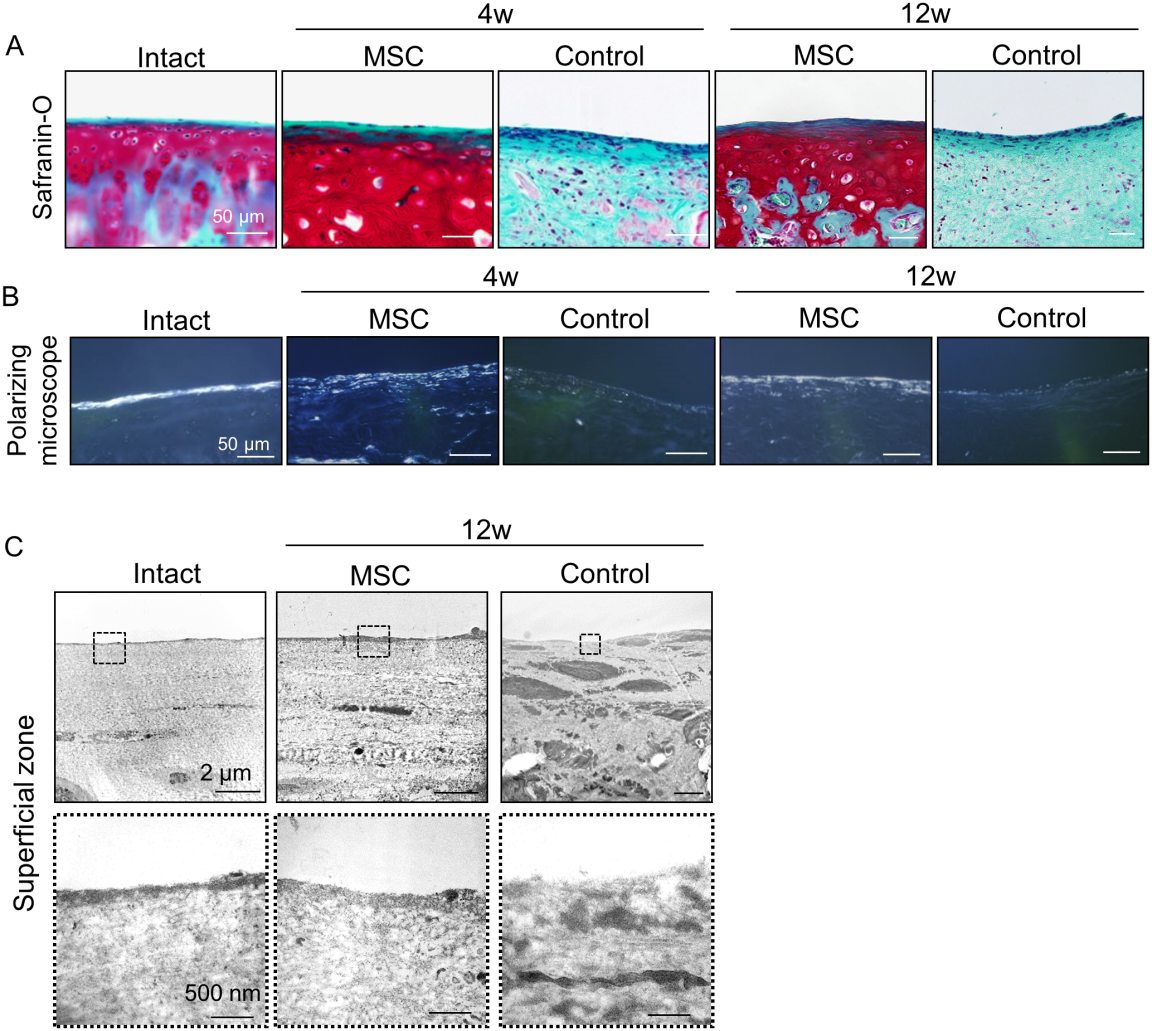
Histological analysis of the superficial zone in regenerated cartilage. (A) Sagittal sections stained with Safranin-O/Fast Green. (B) Sagittal sections unstained for polarizing microscope. (C) TEM images for the superficial zone.

Although flattened cells were observed to be aligned parallel to the joint surface in the superficial zone in all groups in sections stained with Safranin-O/Fast Green (Fig 4A), the brightness of the superficial zone was demonstrated only in the intact and MSC groups by a polarizing microscope (Fig 4B). Transmission electron microscopic analysis at 12 weeks showed developed lamina splendens and flat cells at the surface in the intact and the MSC group, whereas indistinct lamina splendens with a higher number of flat cells was present in the control group (Fig 4C).

### Regenerated Cartilage Immunostained with GFP and Lubricin

**Fig 5:**
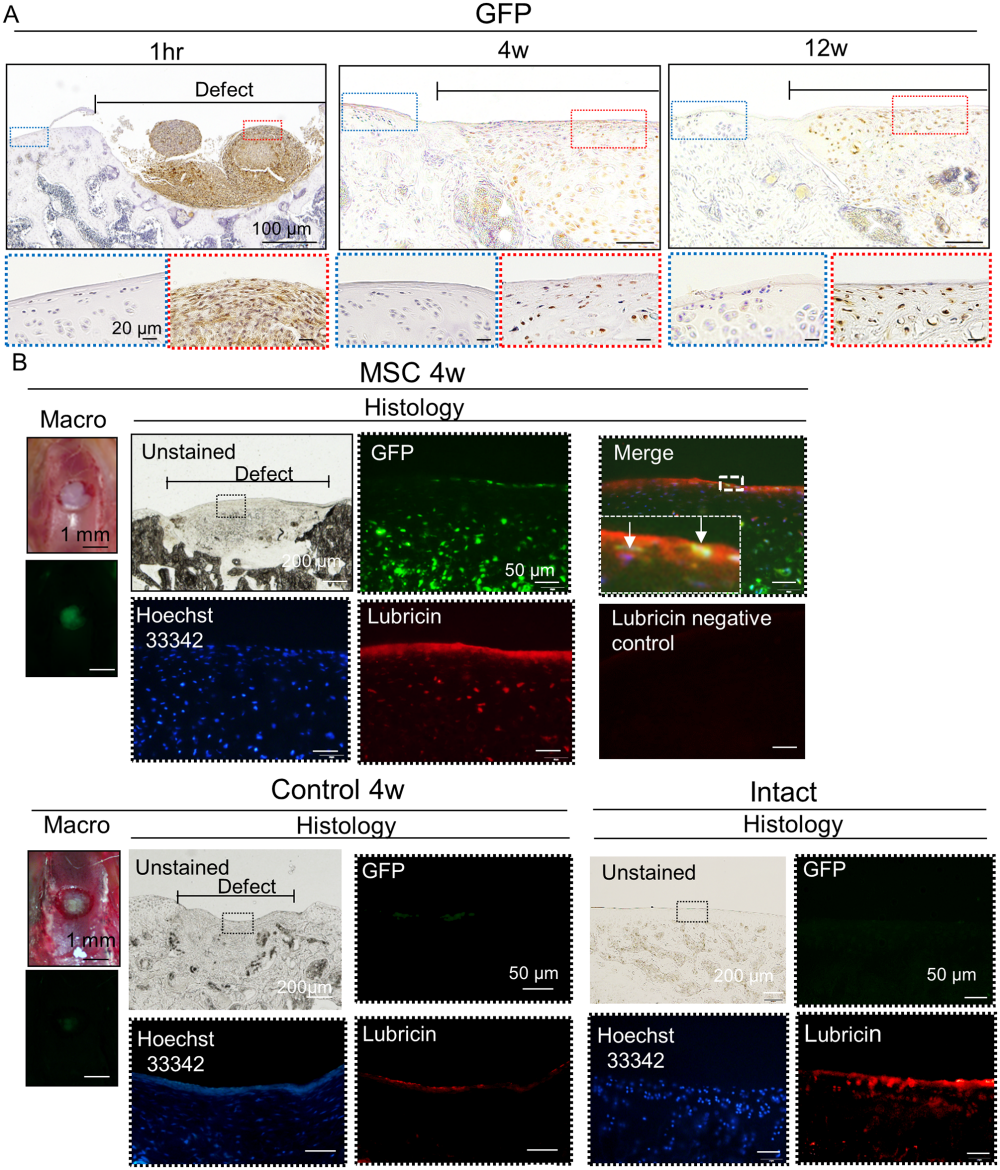
Regenerated cartilage immunostained with GFP and lubricin. (A) Sagittal sections immunostained with GFP. (B) Histological observation under fluorescence for GFP, Hoechst 33342, and lubricin. White arrows indicate both lubricin and GFP positive cells in the superficial zone.

Immunohistological analysis for GFP showed that transplanted aggregates stayed in the osteochondral defect at 1 hour, and GFP cells extended all over the defect at 4 weeks and were still located in the osteochondral defect at 12 weeks, although they were not detected at the adjacent cartilage (Fig 5A). The GFP positive cell rate in the superficial zone was 44 ± 15% at 4 weeks (n = 5), and 47 ± 28% at 12 weeks (n = 5). By fluorescent microscopy at 4 weeks, lubricin expression in the MSC group was apparently higher in the surface of the repaired tissue than that in the control group, and approximately at the same level compared with that in the intact articular cartilage. In the MSC group, GFP positive cells in the superficial zone expressed lubricin protein (Fig 5B).

## Discussion

In this study, cartilage pellets of human MSCs expressed lubricin protein after *in vitro* chondrogenesis. Also, aggregates of human MSCs increased lubricin mRNA expression and showed lubricin protein after hanging drop culture. Furthermore, rat MSCs expressed lubricin protein in the superficial cartilage after transplantation of the aggregates into osteochondral defects in rats. Cartilage derived from MSCs expressed lubricin protein both *in vitro* and *in vivo*. These results indicated that MSCs were able to differentiate into the phenotype of superficial zone chondrocytes that expressed lubricin, and that transplantation of aggregates of MSCs enhanced cartilage regeneration both in the superficial zone and other zones.

The superficial zone of pellets consisted of the flattened cells and was not stained with Safranin-O. These results were similar to those of intact articular cartilage. Previously, Musumeci et al. reported that lubricin expression in the cartilage pellets of adipose MSCs at 21 days was higher than at 7, 14, and 28 days by immunostaining and western blot analysis [47]. In this study, we demonstrated that cartilage pellets had lubricin protein at 21 days by immunostaining. However, lubricin expression was observed at both superficial and deep zones of the pellets, although the extent score for lubricin in the superficial zone was significantly higher than that in the deep zone. Furthermore, lamina splendens was not observed at the surface of pellets. These results were not similar to those of intact articular cartilage, in which lubricin expression is limited to the superficial zone where lamina splendens is shown. Lee et al. mentioned that cartilage pellets *in vitro* could retain less lubricin than articular cartilage [36]. Taken together, the difference of superficial architecture between *in vitro* and *in vivo* should be considered in analysis lubricin expression of cartilage pellets.

Aggregates of human BM MSCs showed different morphological features between the superficial and deep zones; the superficial zone was composed of spindle cells parallel to the surface, whereas the deep zone was composed of round cells. Furthermore, mRNA expressions for Prg4 and Sox9 increased 72 hours after hanging drop culture. Suzuki et al. reported that Prg4 and Sox9 mRNA expressions increased in aggregates of human synovial MSCs [34]. These indicate that only aggregation induces MSCs into the chondrogenic lineage. Prg4 mRNA expression in the aggregates was 106 ± 96 folds with reference to expression in the monolayer culture, while Prg4 mRNA expression in the pellets was 257 ± 224 folds with reference to expression in the monolayer culture. This indicates that Prg4 mRNA expression in the aggregates was lower than that in the cartilage pellets; however, lubricin protein was also detected in the aggregates. These results suggested that aggregates of MSCs by hanging drop culture are useful for articular cartilage regeneration.

In this study, TGF-β3 was used for *in vitro* analysis, while TGF-β3 was not supplemented with culture medium for *in vivo* analysis. Supplementation of TGF-β3 promoted lubricin secretion of superficial zone chondrocytes and synoviocytes *in vitro* [48]. We demonstrated that aggregation enhanced TGF-β3 mRNA expression of BM MSCs. In addition, we were able to observe lubricin expression *in vitro* without TGF-β3 supplementation (data not shown). Based on these results, we thought it is reasonable to detect lubricin expression *in vivo* after transplantation of aggregates by hanging drop culture without TGF-β3 supplementation.

Histologically, the superficial zone was defined as the zone where elongated and flattened chondrocytes were aligned parallel to collagen fibers and to the joint surface with only a staining of Fast Green [16]. In terms of collagen fiber orientation in the superficial zone, polarizing microscope observation revealed that cartilage had uniformity of collagen fiber orientation paralleled to the joint surface as well as the intact cartilage in the MSC group. Transmission electron microscopic analysis also revealed that the MSC group was closer to the intact cartilage than the control group with respect to ultrastructural architecture of lamina splendens of the superficial zone. These results indicated that transplantation of aggregated MSCs promoted regeneration of the superficial zone of articular cartilage.

Transplanted GFP positive cells were observed in the whole osteochondral defect including the superficial zone where lubricin protein expression was also observed at 4 weeks as much as lubricin expression in the intact articular cartilage. Transplanted BM MSCs provided lubricin in the surface of regenerative articular cartilage.

Approximately half of the cells in the superficial zone at 4 weeks were GFP negative cells. The superficial zone is usually covered with synovial fluids. According to our previous reports, after articular cartilage injury, the number of MSCs increased in synovial fluids [49]. Bone marrow cells would also be guided to the lesion. MSCs in synovial fluid and bone marrow cells in addition to transplanted MSCs possibly contributed to the regeneration of the superficial zone of articular cartilage.

**Fig 6:**
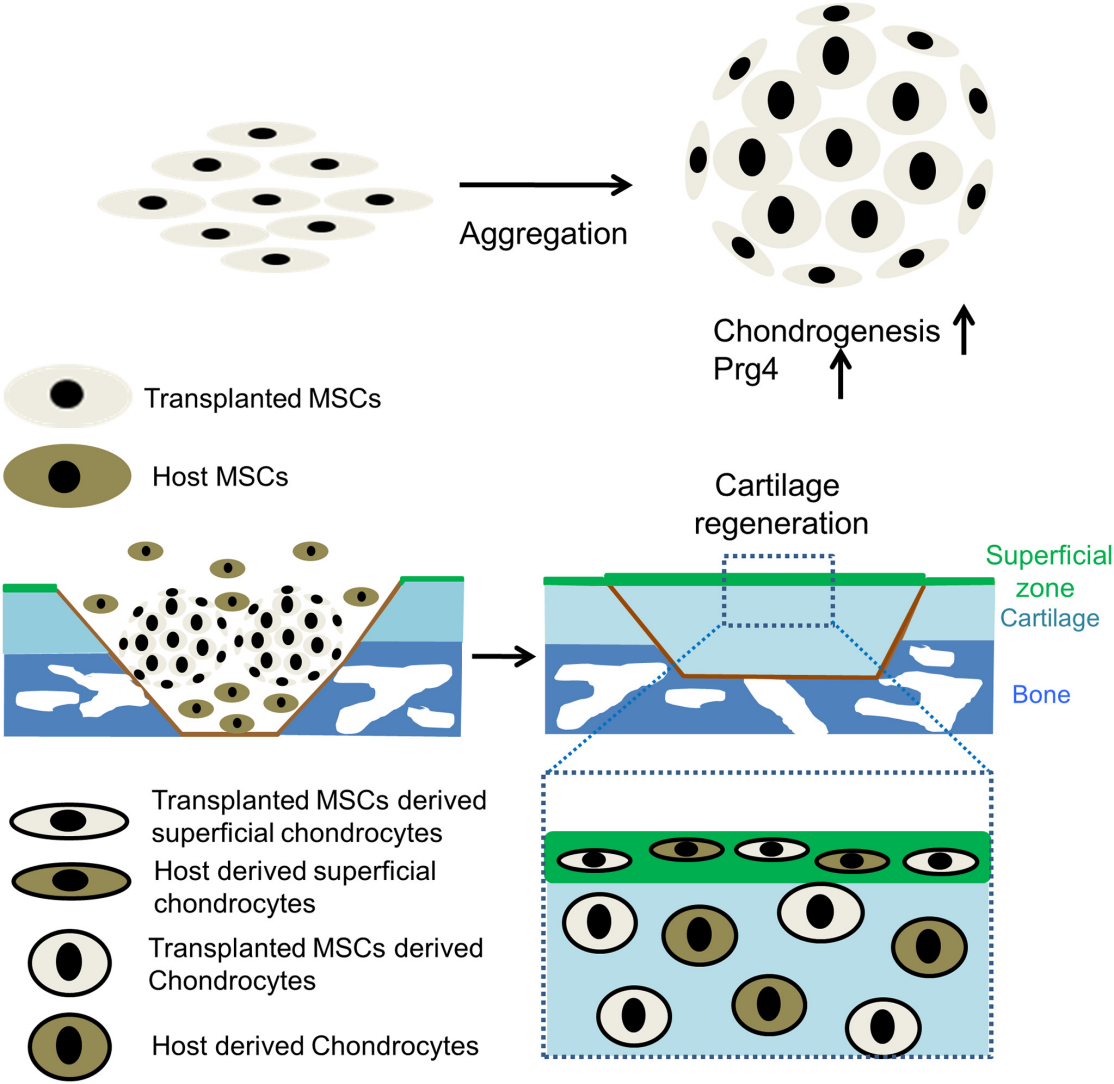
Graphical abstract. Aggregates of MSCs increased level of lubricin expression along with chondrogenesis. After transplantation of aggregates of MSCs to the osteochondral defect, transplanted MSCs and host MSCs contributed to the regeneration of the articular cartilage including superficial zone of articular cartilage. Transplanted MSCs differentiated into superficial chondrocytes expressing lubricin.

According to our *in vitro* and *in vivo* analysis, we summarized the sequence of cellular events during articular cartilage regeneration by aggregated MSCs in Fig 6. MSCs increased the level of Prg4 expression along with chondrogenesis during aggregation by hanging drop culture. After transplantation of aggregates of MSCs to the osteochondral defect, transplanted MSCs and host MSCs contributed to the regeneration of the articular cartilage. Transplanted MSCs differentiated into both chondrocytes and superficial chondrocytes expressing lubricin (Fig 6).

MSCs can be isolated from various mesenchymal tissues, and contain common features [50]. However, there have been several reports showing that variant properties depend on original tissues [31, 51, 52]. Lubricin is secreted by various mesenchymal tissues such as the superficial zone of chondrocytes [53], synoviocytes[54], meniscal cells [55], tenocytes [56], infrapatellar fat pad stromal cells [36], anterior cruciate ligament cells [57], and MSCs derived from BM [28], synovium [36], and adipose tissue [37]. An interesting future study would be to examine whether the effect of aggregated MSCs for promoting lubricin expression and cartilage regeneration depends on their original tissues.

Our results were in accordance with previous reports in which aggregates of chondrocytes or MSCs were used to regenerate articular cartilage [34, 35, 58]. In addition, our current study demonstrated that only aggregation of MSCs induced lubricin expression in MSCs along with chondrogenesis. According to a previous work, fabrication of tissue-engineered cartilage was dependent on lubricin-secreting cells at the surface to attain proper low friction surface property [28]. Taken together, we believe that aggregated MSCs can be clinically useful for articular cartilage regeneration with an appropriate superficial zone in the future.

In regard to limitations of this study, firstly, we used an osteochondral defect model of rats. Self-renewal capacity of articular cartilage and properties of the superficial zone were different between rats and humans [59]. Second, we did not perform a biomechanical test to examine the coefficient friction of the surface of regenerated cartilage. Third, we transplanted aggregated MSCs immediately after we produced an osteochondral lesion, which is not comparable to a clinical situation. Therefore, the results obtained here should be critically considered for future clinical application.

## Conclusion

Cartilage derived from MSCs expressed lubricin protein both *in vitro* and *in vivo*. Transplantation of MSCs regenerated cartilage including the superficial zone in a rat osteochondral defect model.

## Supporting Information

S1 Fig Characteristics of cells derived from human bone marrow. (A) Colony forming potential. (B) Adipogenesis. (C) Calcification. (D) Chondrogenesis. (E) Epitope profile.
(TIF)

S2 Fig Characteristics of bone marrow MSCs from GFP transgenic rats. (A) Light microscope and fluoroscope finding of monolayer culture. (B) Colony forming potential. (C) Adipogenesis. (D) Calcification. (E) Chondrogenesis.
(TIF)

S1 File Supplementary methods for characterization experiments. (DOCX)

S1 Table RT-PCR primer sequences. (DOCX)

S2 Table Histological score for cartilage repair. (DOCX)
