# Supplementary figures and images for: Cartilage Derived from Bone Marrow Mesenchymal Stem Cells Expresses Lubricin In Vitro and In Vivo
Source: PLoS One. 2016 Feb 11;11(2):e0148777. doi: 10.1371/journal.pone.0148777 (PMC4750963; doi:10.1371/journal.pone.0148777)

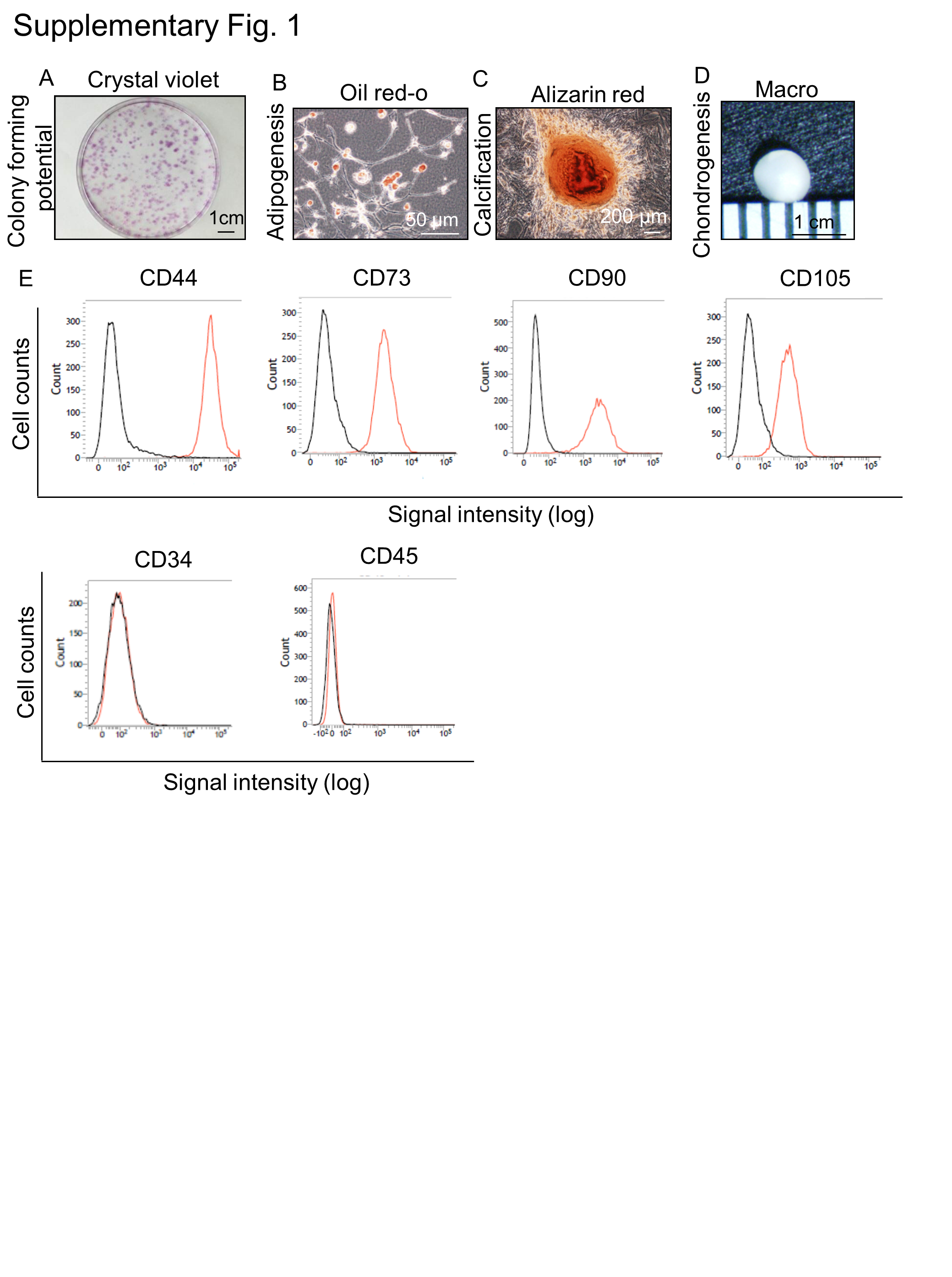

Supplement: S1 Fig — (A) Colony forming potential. (B) Adipogenesis. (C) Calcification. (D) Chondrogenesis. (E) Epitope profile. (TIF) [file pone.0148777.s001.TIF]

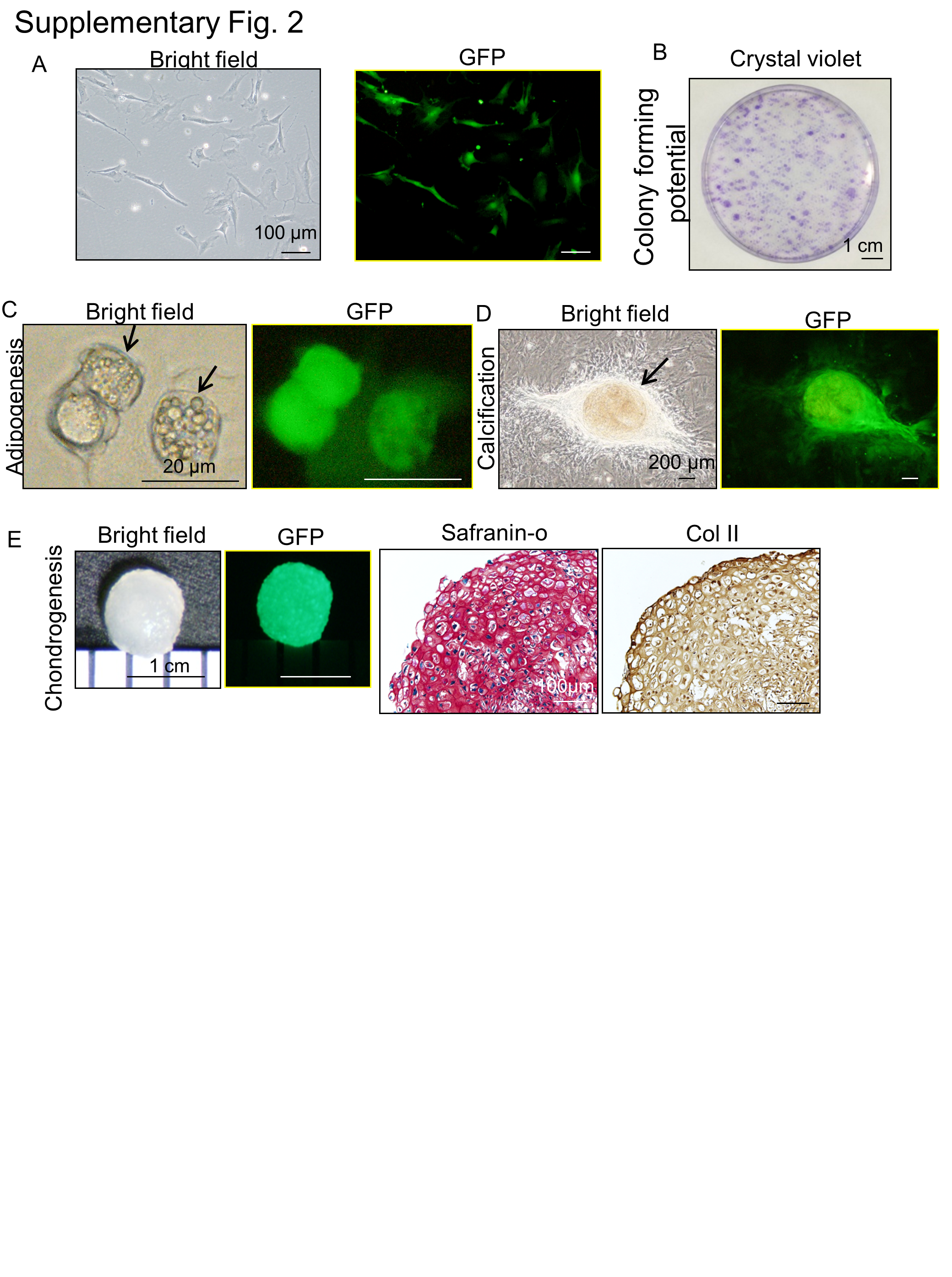

Supplement: S2 Fig — (A) Light microscope and fluoroscope finding of monolayer culture. (B) Colony forming potential. (C) Adipogenesis. (D) Calcification. (E) Chondrogenesis. (TIF) [file pone.0148777.s002.TIF]
