# Supplementary material for: Cartilage Derived from Bone Marrow Mesenchymal Stem Cells Expresses Lubricin In Vitro and In Vivo
Source: PLoS One. 2016 Feb 11;11(2):e0148777. doi: 10.1371/journal.pone.0148777 (PMC4750963; doi:10.1371/journal.pone.0148777)
Supplement: S1 File — (DOCX) [file pone.0148777.s003.docx]

**Supplementary Methods**

**Characterization experiments**

For colony-forming unit assays, one thousand cells from the bone marrow of humans and GFP transgenic rats were plated in 60 cm^2^ dishes, cultured in CCM for 14 days, and stained with 0.5% Crystal Violet (Wako, Osaka, Japan) to observe cell colonies.

For *in vitro* differentiation assays, cells were examined after 21 days in chondrogenic, adipogenic, and osteogenic medium*.* For chondrogenesis, 2.5×10^5^ cells were placed in a 15 mL polypropylene tube (BD Falcon, Bedford, MA) and pelleted by centrifugation at 450 ×g for 10 minutes. The pellets were cultured for 21 days in chondrogenic medium, which contained 10 ng/mL transforming growth factor-𝛽3 (TGF-𝛽3: R&D Systems Inc., Minneapolis, MN), and 100 nM dexamethasone (Sigma-Aldrich, St. Louis, MO). For histological analysis, the pellets were embedded in paraffin, cut into 5 μm sections, and stained with Safranin-O/Fast Green.

For adipogenesis, the cells were cultured in the adipogenic medium that consisted of a CCM supplemented with 0.5 μM dexamethasone (Sigma-Aldrich), 0.5 mM isobutylmethylxanthine (Sigma-Aldrich), and 50 μM indomethacin (Wako). After 21 days, the adipogenic cultures were stained with 0.3% Oil Red-O solution.

For mineralization, the cells were cultured in CCM supplemented with 100 nM dexamethasone, 10 mM 𝛽-glycerophosphate, and 50 μM ascorbic acid. After 21 days, the dishes were washed with PBS twice and fixed in 10% formalin for 5 minutes and stained with 2% Alizarin Red solution (pH 4.1; Sigma-Aldrich) for 2 minutes.^14^

For analyzing expression of surface markers, cells at passage 2 were harvested 7 days after plating. 1×10^5^ cells were suspended in 1 ml FACS buffer (0.2% BSA and 0.09% sodium azide with PBS) containing 400 ng/mL phycoerythrin-(PE-) coupled antibodies against CD34, CD44, CD45, CD73, CD90 and CD105 (all from BD Bioscience, San Diego, CA). Control staining was performed with a directly labeled isotype matched monoclonal antibody. After incubation for 1 hour at 4°C, the cells were washed with PBS and resuspended in 1 ml FACS buffer for analysis. Cell fluorescence was evaluated by flow cytometry in a FACSVerse instrument (BD); data were analyzed using FACSite software (BD).
