# Supplementary material for: Cartilage Derived from Bone Marrow Mesenchymal Stem Cells Expresses Lubricin In Vitro and In Vivo
Source: PLoS One. 2016 Feb 11;11(2):e0148777. doi: 10.1371/journal.pone.0148777 (PMC4750963; doi:10.1371/journal.pone.0148777)
Supplement: S1 Table — (DOCX) [file pone.0148777.s004.docx]

**Supplementary Table 1.**

**RT-PCR primer sequences**

| **Primer name** | | **Sequences** | **Probe No.** |
| --- | --- | --- | --- |
| **Prg4** | forward | 5’-CTGGCCTGAATCTGTGTATTTTT-3’ | 87 |
|  | backward | 5’-TTCTTCCAGGGCACTTCTGT -3’ |  |
| **Sox9** | forward | 5’-GTACCCGCACTTGCACAAC-3’ | 61 |
|  | backward | 5’-TCGCTCTCGTTCAGAAGTCTC-3’ |  |
| **Aggrecan** | forward | 5’-CTGGAAGTCGTGGTGAAAGG-3’ | 21 |
|  | backward | 5’-TCGAGGGTGTAGCGTGTAGA-3’ |  |
| **Tgfβ3** | forward | 5’-AAGAAGCGGGCTTTGGAC-3’ | 38 |
|  | backward | 5’-CGCACACAGCAGTTCTCC-3’ |  |
| **Bmp2** | forward | 5’-CGGACTGCGGTCTCCTAA-3’ | 49 |
|  | backward | 5’-GGAAGCAGCAACGCTAGAAG-3’ |  |
| **β-actin** | forward | 5’-ATTGGCAATGAGCGGTTC-3’ | 11 |
|  | backward | 5’-TGAAGGTAGTTTCGTGGATGC-3’ |  |
