# Supplementary material for: Cartilage Derived from Bone Marrow Mesenchymal Stem Cells Expresses Lubricin In Vitro and In Vivo
Source: PLoS One. 2016 Feb 11;11(2):e0148777. doi: 10.1371/journal.pone.0148777 (PMC4750963; doi:10.1371/journal.pone.0148777)
Supplement: S2 Table — (DOCX) [file pone.0148777.s005.docx]

**Supplementary Table 2.**

| **Histological score for cartilage repair** | | | | |
| --- | --- | --- | --- | --- |
| **Category** | |  |  | **Score** |
| A. Cell morphology | |  |  |  |
| Hyaline cartilage | |  |  | 4 |
| Mostly hyaline cartilage | | |  | 3 |
| Mostly fibrocartilage | |  |  | 2 |
| Mostly non-cartilage | |  |  | 1 |
| Non-cartilage only | |  |  | 0 |
| B. Matrix-staining (metachromasia) | | | |  |
| Normal (compared with adjacent cartilage) | | | | 3 |
| Slightly reduces | |  |  | 2 |
| Markedly reduced | |  |  | 1 |
| No metachromatic stain | | |  | 0 |
| C. Surface | |  |  |  |
| Smooth (>3/4) | |  |  | 3 |
| Moderate (1/2 to 3/4) | |  |  | 2 |
| Irregular (1/4 to 1/2) | |  |  | 1 |
| Severely irregular (<1/4) | | |  | 0 |
| D. Thickness of cartilage | | |  |  |
| 2/3 to 4/3 |  |  |  | 3 |
| 5/3 to 4/3 |  |  |  | 2 |
| 1/3 to 2/3 or >5/3 | |  |  | 1 |
| <1/3 |  |  |  | 0 |
| E. Integration of donor with host cartilage | | | |  |
| Both edges integrated | | |  | 2 |
| One edge integrated | |  |  | 1 |
| Neither edge integrated | | |  | 0 |
| Total maximum | |  |  | 15 |
